# Evaluation of the Diagnostic Ability of Optical Enhancement System in Early Gastric Cancer Demarcation

**DOI:** 10.1155/2016/2439621

**Published:** 2016-09-28

**Authors:** Misato Nagao, Jun Nishikawa, Ryo Ogawa, Sho Sasaki, Munetaka Nakamura, Junichi Nishimura, Atsushi Goto, Shinichi Hashimoto, Takeshi Okamoto, Masato Suenaga, Yoshihiko Hamamoto, Isao Sakaida

**Affiliations:** ^1^Department of Gastroenterology & Hepatology, Yamaguchi University Graduate School of Medicine, Ube, Japan; ^2^Department of Laboratory Science, Yamaguchi University Graduate School of Medicine, Ube, Japan; ^3^Department of Biomolecular Engineering Applied Molecular Bioscience, Yamaguchi University Graduate School of Medicine, Ube, Japan

## Abstract

This study aimed to evaluate the utility of optical enhancement (OE) in early gastric cancer demarcation. Twenty lesions of early gastric cancer were examined by PENTAX endoscopy system with OE-1 and OE-2 functions. The areas of tumor demarcation identified by 12 evaluators (6 novice and 6 experienced) were compared to the corresponding correct areas determined by postoperative histopathology findings. The misdiagnosed scores that were the sums of false-positive and false-negative areas were compared. Color of one hundred pixels from the inside of the cancerous area and the outside of the cancerous area was expressed as three-dimensional RGB component vectors. The mean vectors and covariance matrixes were calculated and the Mahalanobis distance, indicative of color differences between two areas, was tested. Comparisons of the misdiagnosed score revealed that OE-1 was preferred over WL-1 for gastric cancer demarcation for all 12 evaluators (*p* = 0.008) and in novice evaluators (*p* = 0.026). OE-2 was not significantly different from WL-2 in all cases. OE-1 images gave significantly larger Mahalanobis distances, indicative of color differences, than WL-1 images (*p* = 0.002). It was demonstrated that the OE Mode 1 has a significant advantage over the white light mode in demarcation of early gastric cancer.

## 1. Introduction

The diagnosis of early gastric cancer relies on identification of slight changes in mucosal color and depression/elevation, and, thus, it is difficult to achieve. In recent years, image-enhanced endoscopy (IEE) has been advancing and is superior to white light observation. Narrowband imaging (NBI) is an optical digital imaging technique wherein two selected wavelengths (415 ± 30 nm and 540 ± 30 nm) enhance the structural aspects of the surface of the mucosa and existing vessels. Its utility in the diagnosis of early gastric cancer and in demarcation of gastric cancer margins has already been demonstrated [1–4]. Flexible spectral imaging color enhancement (FICE) is another type of IEE based on spectral image processing technology. The FICE system provides high-contrast images by enhancement of color differences between the tumor and normal mucosa [5, 6].

Two optical enhancement (OE) modes were recently developed by PENTAX. The OE Mode 1 (OE1) uses light emission at 415 nm and 540 nm, which are suitable for visualizing blood vessels on the mucosal surface and in the submucosa, respectively, enhancing the contrast between vessels in superficial layers and those in the deep layers. On the other hand, the OE Mode 2 (OE2) uses red light emission as well as emission at 415 nm and 540 nm to increase the overall brightness of the image, thereby obtaining images closer to the corresponding white light images.

This study employed the twin mode, which enables simultaneous display of a white light (WL) and the corresponding OE image, and compared OE images with the corresponding WL images in order to evaluate the utility of OE in the demarcation of early gastric cancer.

## 2. Materials and Methods

### 2.1. Subjects

Twenty lesions in 20 cases of early gastric cancer treated between November 2013 and April 2014 at Yamaguchi University Hospital were examined. Histopathology confirmed the diagnosis in all cases.  Table 1 shows the clinicopathological characteristics of subjects. Seventeen lesions were treated by endoscopic submucosal dissection, while three were surgically excised. In accordance with the macroscopic classification, 10 lesions were elevated, while the other 10 were depressed. Eighteen lesions were differentiated, while 2 were undifferentiated gastric adenocarcinomas. We surgically removed one undifferentiated cancer. The other case was treated by ESD because the patient had severe complications. Ten lesions were confined to the mucosa and the other 10 were confined to the submucosa in accordance with the classification by the depth of tumor invasion. Three of the 10 submucosal invasive tumors were removed surgically. The other 7 patients underwent ESD. Based on the pathological diagnosis after ESD, 4 patients had additional surgery. One patient was followed closelywithout additional surgery because of shallow submucosal invasion. Two patients are also being followed closely because they could not undergo surgery due to advanced age and complications. Endoscopic procedures were explained, and informed consent was obtained from all subjects before examination. This study was approved by the Ethics Committee of Yamaguchi University Hospital.

### 2.2. OE and Twin Mode

A PENTAX EG29-i10N upper gastrointestinal endoscope (HOYA, Tokyo, Japan) and an EPK-i7000 processor (HOYA, Tokyo, Japan) were used. The twin mode that enables simultaneous display of a white light image and the corresponding endoscopic image taken with either the OE Mode 1 or the OE Mode 2 (OE-1 image or OE-2 image, resp.) was used to obtain two paired still images for each lesion: an OE-1 image with the corresponding WL image (WL-1) and an OE-2 image with the corresponding WL image (WL-2). Paired still images were then divided into WL and OE images, yielding four images for each of the 20 lesions (a total of 80 images). Each still image was pasted onto a Microsoft PowerPoint slide as background, in order to prevent unintentional movement and resizing of the image by evaluators. Eighty images on 80 slides were randomly sorted to eliminate the influence of the displaying order of WL-1, OE-1, WL-2, and OE-2 on the outcomes of evaluation.

### 2.3. Evaluators

Evaluators were 12 physicians blinded to the information about the subjects. Six were junior residents who completed a 2-month training period in the Department of Gastroenterology and Hepatology, Yamaguchi University Hospital (novice evaluators), and the other 6 were specialists certified by the Japan Gastroenterological Endoscopy Society who had evaluated ≥3,000 cases (experienced evaluators).

### 2.4. Evaluation of the Demarcation of Gastric Carcinomas

A total of 80 still images of 20 lesions were evaluated on PowerPoint slides. Briefly, evaluators circled the suspected area where a gastric cancer was exposed on the mucosal surface using one of PowerPoint graphics function, namely, the freehand function. The areas of cancer demarcation identified by evaluators were compared to the corresponding areas of correct cancer demarcation determined by postoperative histopathology findings. Evaluators were asked to examine images in sorted order, and reexamination of once-examined images was strictly prohibited, thereby minimizing the influence of the order of still images on evaluation results. There was no time limit for completion of demarcation on one image.

A specialist certified by the Japan Gastroenterological Endoscopy Society (Jun Nishikawa) examined endoscopic images with corresponding macroscopic and histopathology findings and then similarly circled a cancer-exposing area on individual images. These areas served as correct areas. Images with the areas of cancer demarcation identified by evaluators (test images) or the corresponding areas of correct areas (reference images) were converted to PNG files and then to PPM files for image processing. All images were binarized by filling the inside of the circled area in black, while the outside was in white. Test images were overlaid on the corresponding reference images to obtain the false-positive areas (black on the test image and white on the reference image, incorrectly judged as cancerous) and the false-negative areas (white on the test image and black on the reference image, incorrectly judged as noncancerous). The sum of false-positive and false-negative areas was referred to as the misdiagnosed area. Next, we tried to incorporate the influence of the proportion of evaluators who diagnosed a pixel within the misdiagnosed area incorrectly. Briefly, in each of three evaluator group (all 12 evaluators (*n* = 12); novice evaluators (*n* = 6) and experienced evaluators (*n* = 6)), all misdiagnosed areas were overlaid, and pixels within the misdiagnosed areas were weighed using the proportion of evaluators who diagnosed those pixels incorrectly. A pixel that was incorrectly identified cancerous or noncancerous by all evaluators was provided with the weight of 1. This yielded the following equation: (the weight of each pixel within the misdiagnosed areas) = (number of evaluators who diagnosed that pixel incorrectly)/(number of total evaluators in the group). The total sum of weight of an individual pixel within the misdiagnosed areas on a still image was referred to as the misdiagnosed score. In this study, a smaller misdiagnosed score was considered to indicate superiority of the image in the demarcation of a gastric cancer.

### 2.5. Evaluation Using the RGB Colorimetric System

Using 80 still images of 20 lesions, 100 pixels each were randomly selected from the inside of the cancerous area and the outside of the cancerous area per image. Selected pixels (200 pixels/image) were expressed as three-dimensional RGB component vectors. The mean vectors (*μ*1) and covariance matrix (Σ1) were estimated using 100 three-dimensional vectors in the cancerous area, and, similarly, the mean vectors (*μ*2) and covariance matrix (Σ2) were estimated using 100 three-dimensional vectors in the noncancerous area on the image. The Mahalanobis distances, indicative of color differences between cancerous and noncancerous areas, were obtained for WL-1, OE-1, WL-2, and OE-2, and they were referred to as DWL-1, DOE-1, DWL-2, and DOE-2, respectively [7]. Differences in the Mahalanobis distances between WL-1 and OE-1 (DOE-1/DWL-1) and between WL-2 and OE-2 (DOE-2/DWL-2) were calculated for each of the 20 lesions. Paired *t*-test was used for statistical analysis.

### 2.6. Histogram Display of the False Demarcation Area

Weighted pixels within the misdiagnosed area were visualized to demonstrate the frequency of incorrect demarcation. Misdiagnosed areas by 12 evaluators were overlaid, and pixels that were incorrectly identified by two or more evaluators were expressed in colors: blue for the false-negative areas and green for the false-positive areas. Darker colors indicated the greater frequency of incorrect demarcation. Images with smaller colored areas in a lighter color were considered to be better in terms of gastric cancer demarcation.

### 2.7. Statistics

Data were evaluated by the *t*-test for statistical significance (*p* < 0.05).

## 3. Results

### 3.1. Comparisons of Areas of Misdiagnosis

**Figure 1:**
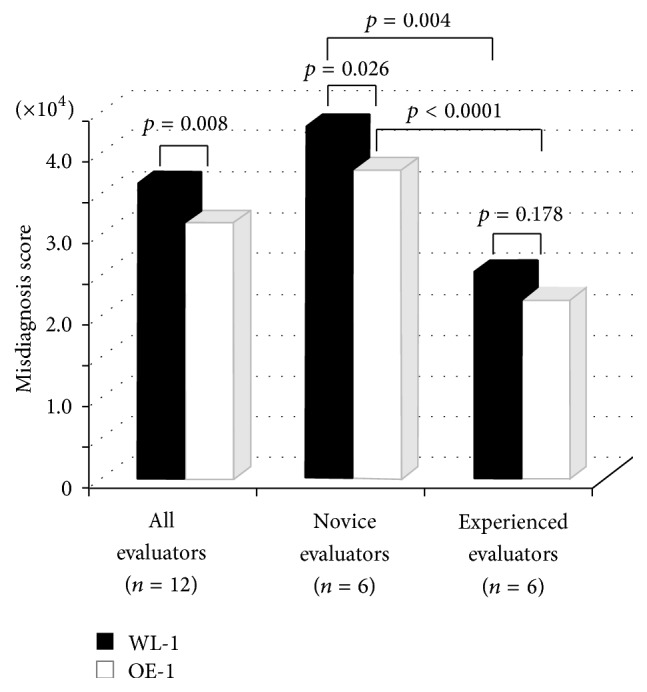
Comparative analysis of misdiagnosed score on WL-1 and OE-1 images. Compared with WL-1 images, misdiagnosis score on OE-1 images was smaller for the 6 novice evaluators and for all 12 evaluators. No significant difference was observed among the 6 experienced evaluators.

**Figure 2:**
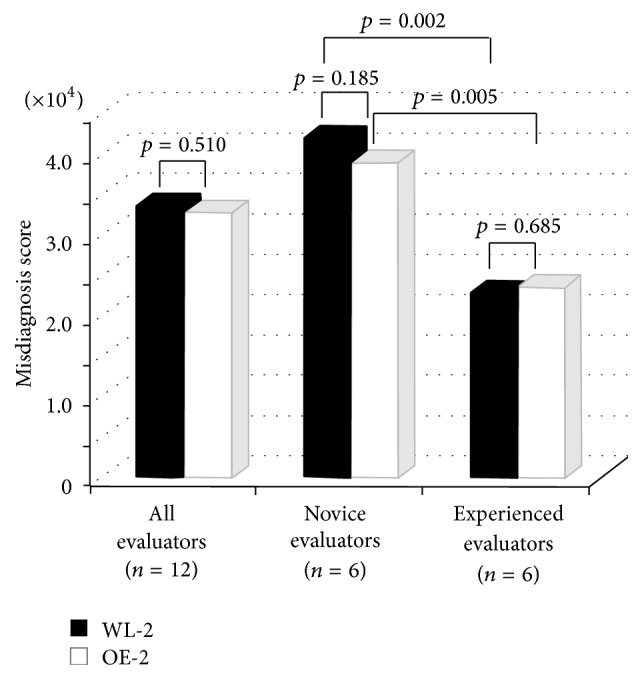
Comparative analysis of misdiagnosed score on WL-2 and OE-2 images. No significant difference was observed between the WL-2 and OE-2 images of different lesions.

Overall, the misdiagnosed score by 12 gastroenterologists in 20 lesions was smaller with OE-1 images than with WL-1 images, and this was notable for the six novice gastroenterologists. The misdiagnosed score did not differ significantly between OE-1 and WL-1 images for experienced gastroenterologists, and the misdiagnosis score was smaller for experienced evaluators than for novice evaluators regardless of the images displayed (Figures 1 and 2). The OE-1 mode improved the demarcation of elevated lesions among all 12 evaluators (28686 versus 23458, *p* = 0.008) and novice evaluators (36534 versus 29245, *p* = 0.017). Similarly, the OE-1 mode improved the demarcation of mucosal discoloration among all 12 evaluators (28224 versus 20519, *p* = 0.014) and demarcation of differentiated adenocarcinomas among novice evaluators (44059 versus 38577, *p* = 0.041) and all 12 evaluators (36547 versus 32182, *p* = 0.022). Furthermore, the OE-1 mode improved the demarcation of the lesion located in middle stomach among experienced evaluators (31239 versus 24382, *p* = 0.025) and all 12 evaluators (40418 versus 33042, *p* = 0.003) and the demarcation of intramucosal cancer among novice evaluators (40752 versus 31454, *p* = 0.026) and all 12 evaluators (32980 versus 24805, *p* = 0.007) (Table 2).

As for OE-2 and WL-2 images, the misdiagnosed score was insignificant in all groups (Figure 2).

### 3.2. Evaluation Using the RGB Color System

**Figure 3:**
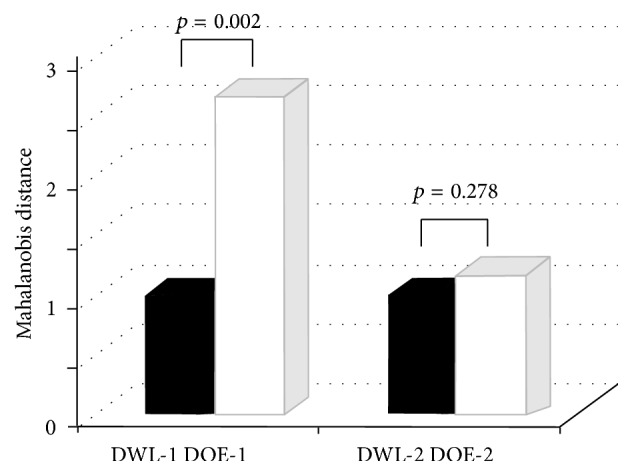
Evaluation using the RGB color system. Compared with WL-1, Mahalanobis distance was significantly larger on OE-1 images. No significant difference was observed between OE-2 and WL-2 findings.

The Mahalanobis distance was used to express the color difference in RGB three-dimensional vectors between cancerous and noncancerous areas. The comparisons of DOE-1 and DWL-1, which are the Mahalanobis distance in OE-1 and WL-1 images, respectively, revealed that DOE-1 was significantly larger than DWL-1 (DOE-1/DWL-1 = 2.67; *p* = 0.002). In other words, compared with WL-1, the color of cancerous and noncancerous areas differed significantly on OE-1 images, making the differentiation of the two areas easier. On OE2 images, the differential diagnosis of cancerous and noncancerous areas was difficult because of little difference in color (DOE-2/DWL-2 = 1.17; *p* = 0.278) (Figure 3).

### 3.3. Display of Histograms

**Figure 4:**
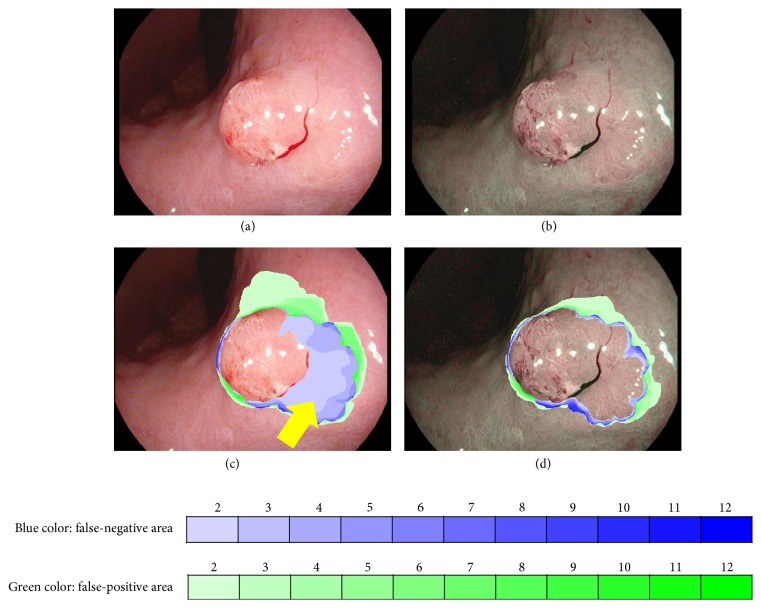
Histogram display of the misdiagnosed area. Early gastric cancer 0-I + IIa at the angle of the lesser curvature. (a) WL-1 images; (b) OE-1 images; (c) WL-1 image histograms; and (d) OE-1 image histograms. Blue color depicts the false-negative area, and green color depicts the false-positive area. The areas with darker colors indicate a higher incidence of misdiagnosis. In this study, areas of misdiagnosis were smaller and the color was lighter on OE-1 images than on WL-1 images.

We displayed the histograms of WL-1 and OE-1 images to clarify which images improve the demarcation of each lesion type and in what areas diagnostic errors occurred frequently. The WL image of Figure 4(a) showed a polypoid lesion with red color in the lesser curvature of the gastric angle. The flat elevation at the base of the polypoid lesion was considered as a false-negative area on WL image (Figure 4(c), yellow arrow). The brown color over the flat elevation was enhanced on OE-1 images (Figure 4(b)), leading to an accurate diagnosis (Figure 4(d)).

## 4. Discussion

Preoperative diagnosis of tumor demarcation is becoming increasingly important with the incorporation of endoscopic treatment for gastric cancer. Compared with chromoendoscopy, the diagnostic accuracy of a cancer demarcation in early gastric cancer is superior in magnifying endoscopy with NBI [8, 9]. Though NBI observation is effective with magnifying image, we showed that the demarcation accuracy of early gastric cancer was higher in OE-1 without magnifying imaging compared with WL imaging. OE-1 uses white light components in addition to narrowband light at the 415 nm and 540 nm wavelengths. NBI does not use white light components. Because images by OE-1 are brighter than those by NBI, we believe that OE-1 has the ability to determine tumor demarcation without magnifying imaging. FICE is based on spectral image processing technology [10]. It was reported that FICE has the ability of diagnosis of demarcation line of early gastric cancer without magnification [6, 11, 12]. Blue laser imaging (BLI) utilizes two monochromatic lasers (410 and 450 nm). A 410 nm laser visualizes vascular microarchitecture, and a 450 nm laser provides white light by excitation [13]. The linked color imaging (LCI) method is based on a BLI-bright image with additional image processing that enhances color separation of the red color to depict red and white colors more vividly [14]. The ability to diagnose the demarcation line of early gastric cancer without magnification has not been fully evaluated by using BLI and LCI.

The diagnostic capability of experienced evaluators was clearly superior to that of novice evaluators. The experienced evaluators were more than capable of demarcating gastric cancer under WL. This study showed that the demarcation accuracy of gastric cancer of novice evaluators was improved by OE-1. We used the RGB color system to investigate whether OE-1 emphasizes the color difference between cancerous and noncancerous areas. The results showed that the difference in RGB three-dimensional vectors between cancerous and noncancerous areas was greater in OE-1 compared with WL-1, with OE-1 enhancing the difference in color. This study is the first to display color difference between cancerous and noncancerous areas using the RGB color system in image-enhanced endoscopy.

OE-1 images are similar to NBI images because of the close wavelengths used in the two systems. Therefore, Japanese novice gastroenterologists may be more familiar with OE-1 images, leading to better results. The demarcation accuracy of early gastric cancer did not differ significantly between OE-2 and WL-2, suggesting that it may be necessary to get used to OE-2. We used the twin mode images; that is, the endoscopic image enhancement system enables the concurrent display and comparison of OE and WL images on the monitor in the present evaluation. Light intensity needed for the two mode was sometimes different; the halation was observed in some close-up WL images. It is necessary to establish an imaging and viewing protocol appropriate for the light intensity in each mode.

No guidelines for the accurate diagnosis of demarcation in early gastric cancer have been established. Previous studies have reported the usefulness of magnifying endoscopy for tumor demarcation [15, 16]; however, methods used in these studies are most likely influenced by the ability of evaluators and lack objectivity. We could perform a highly objective comparison of diagnostic capability by asking multiple physicians to evaluate twin mode images. We believe that our method for evaluating tumor demarcation must be useful for understanding the utility of newly developed image-enhanced endoscopy system. We first developed the misdiagnosed score to evaluate the area of misdiagnosis. We can easily know the characteristic of lesions that many evaluators misdiagnosed. In Figure 4, the extent of tumor demarcation which was located at the base of main protrusion was identified by OE-1 mode because the color of the area turned brownish. Four or 5 of 12 evaluators misdiagnosed as false-negative area. To improve diagnostic capability of cancer margin, our method could be useful educationally for especially novice endoscopists.

The limitation of this study that examined 20 patients with early gastric cancer at a single institution is the small number of cases. Therefore, we plan to increase the number of patients and compare OE and NBI images in the future.

The findings of this study revealed that OE-based endoscopic imaging improves the demarcation accuracy of early gastric cancer compared with white light endoscopic imaging.

## Figures and Tables

**Table 1:** Clinicopathological features of the early gastric carcinomas.

Patient age (years)	Mean	73.6
Sex	Male	18
	Female	2
Location	Upper	3
	Middle	13
	Lower	4
Lesion diameter	<10 mm	6
	10–20 mm	11
	>20 mm	3
Macroscopic type	Elevated type	10
	Depressed type	10
Color	Reddish	14
	Normal-colored	2
	Discolored	4
Tumor differentiation	Differentiated	18
	Undifferentiated	2
Invasion depth	Mucosal layer	10
	Submucosal layer	10

**Table 2:** Misdiagnosed score of WL-1 and OE-1 images by macroscopic type, color, tumor differentiation, location, and invasion depth.

		All evaluators (*n* = 12)			Novice evaluators (*n* = 6)			Experienced evaluators (*n* = 6)		
		WL-1	OE-1	*p* value	WL-1	OE-1	*p* value	WL-1	OE-1	*p* value
Macroscopic type	Elevated	28686	23458	0.008	36534	29245	0.017	18171	15850	0.189
	Depressed	43665	39362	0.168	49952	46492	0.379	32642	28019	0.360
Color	Reddish	37293	32915	0.067	44830	40431	0.126	25706	21807	0.287
	Normal-colored	44250	42655	0.528	47885	49876	0.280	37033	33458	0.132
	Discolored	28224	20519	0.014	35368	22896	0.071	18544	16619	0.507
Differentiation	Differentiated	36547	32182	0.022	44059	38577	0.041	25340	22644	0.331
	Undifferentiated	32832	24459	0.239	35901	31491	0.285	26002	15555	0.116
Location	Upper	16819	15951	0.731	23769	22438	0.715	8669	7066	0.247
	Middle	40418	33042	0.003	45736	39228	0.061	31239	24382	0.025
	Lower	36903	37702	0.758	49746	45025	0.345	19004	25133	0.477
Invasion depth	Mucosal layer	32980	24805	0.007	40752	31454	0.026	22127	15661	0.089
	Submucosal layer	39370	38015	0.413	45733	44283	0.545	28686	28209	0.895

OE-1: optical enhancement Mode 1, WL-1: white light 1.
